# Signaling between mammalian adiponectin and a mosquito adiponectin receptor reduces *Plasmodium* transmission

**DOI:** 10.1128/mbio.02257-23

**Published:** 2023-12-11

**Authors:** Yu-Min Chuang, Helen Stone, Selma Abouneameh, Xiaotian Tang, Erol Fikrig

**Affiliations:** 1 Section of Infectious Diseases, Department of Internal Medicine, School of Medicine, Yale University, New Haven, Connecticut, USA; The Ohio State University, Columbus, Ohio, USA

**Keywords:** *Plasmodium*, adiponectin, mosquito, adiponectin receptor, lipophorin

## Abstract

When a female mosquito engorges on a mammalian host, components of the blood meal can affect mosquito fitness and indirectly influence pathogen infectivity. We demonstrate that mammalian adiponectin, ingested during *Anopheles gambiae* blood feeding, co-localizes within mosquito midguts and decreases *Plasmodium* infection in the vector. Transcriptomic and RNAi studies reveal that the *A. gambiae* adiponectin receptor is involved in downregulating lipophorin, a lipid transporter that is important for egg development and *Plasmodium* infection in mosquitoes. These studies characterize a cross-phyla interaction between the mammalian host and *A. gambiae* that negatively impacts *Plasmodium* survival in its arthropod vector.

**IMPORTANCE:**

When a female mosquito takes a blood meal from a mammalian host, components of the blood meal can affect mosquito fitness and indirectly influence pathogen infectivity. We identified a pathway involving an *Anopheles gambiae* adiponectin receptor, which, triggered by adiponectin from an incoming blood meal, decreases *Plasmodium* infection in the mosquito. Activation of this pathway negatively regulates lipophorin expression, an important lipid transporter that both enhances egg development and *Plasmodium* infection. This is an unrecognized cross-phyla interaction between a mosquito and its vertebrate host. These processes are critical to understanding the complex life cycle of mosquitoes and *Plasmodium* following a blood meal and may be applicable to other hematophagous arthropods and vector-borne infectious agents.

## INTRODUCTION

*Plasmodium* species start their sporogonic cycle when a female mosquito takes a blood meal from an infected vertebrate host. After the blood meal, gametocytes generate zygotes, which transform into ookinetes in the mosquito midgut. Ookinetes develop into oocysts, which subsequently release sporozoites to start another infection cycle. *Plasmodium* utilize nutrients from the mosquito for development, and at the same time, mosquitoes use diverse mechanisms to limit the impact of *Plasmodium* on their fitness (1, 2). During or after blood meals, mammalian proteins, complement factors, or cytokines can interact with *Plasmodium* and mosquitoes to affect the infectivity of gametocytes (3, 4). Cytokines and reactive nitrogen species within the lumen of the mosquito midgut have an impact, which may be either direct or indirect, on the development of *Plasmodium* (3, 5, 6). As one example, after a blood meal, mammalian transforming growth factor β1 can activate the synthesis of nitric oxide in the midgut of mosquitoes, thereby affecting *Plasmodium* development (5).

The reproductive fitness of mosquitoes and their lifespan—directly or indirectly—influence susceptibility to *Plasmodium* and contribute to the burden of malaria in endemic areas (7). 20-Hydroxyecdysone hormone (20E) is one of the major pathways that can affect egg development after a blood meal and silencing of 20E affects *Plasmodium* development in mosquitoes (8–10). Two downstream genes in the 20E pathway, *lipophorin* (Lp) and *vitellogenin* (Vg), serve as important effector genes for lipid transport and metabolism after the blood meal and regulate vitellogenesis and oogenesis (2, 9, 11–16). Silencing of *lipophorin* or *Vg* leads to fewer *Plasmodium* in the mosquitoes (13, 17). Based on all this information, lipid transport and lipid utilization serve as an important link between oogenesis and *Plasmodium* development inside the mosquito.

Adiponectin, the adipocyte complement-related protein of 30 kDa (or Acrp30), regulates metabolism, alters insulin sensitivity, and affects inflammation in different species (18–20). The effects of adiponectin depend, in part, on binding to adiponectin receptors via a globular C1q domain (19, 21). There are two mammalian adiponectin receptors, AdipoR1 and AdipoR2 (22). Adiponectin receptors have seven transmembrane domains with an internal N terminus and an external C terminus (22). *AdipoR1-* and *AdipoR2-*deficient mice have glucose intolerance and increased triglyceride levels, demonstrating that the adiponectin pathway regulates glucose and lipid homeostasis (18, 23). An adiponectin receptor homologue in *Drosophila melanogaster* modulates insulin secretion and also controls both glucose and lipid metabolism, despite the absence of an identified adiponectin homologue in fruit flies (24). Recently, our group demonstrated that *Ixodes scapularis* ticks have an adiponectin receptor-like gene (*ISARL*) and ISARL both regulates phospholipid metabolism and facilitates *Borrelia burgdorferi* colonization of the tick (25). These interactions are mediated through an *I. scapularis* complement C1q-like protein (25).

In the present study, we characterize an adiponectin receptor (*AdpR, AGAP004486*) from *Anopheles gambiae* and its role when mosquitoes take a blood meal. Transcriptomic analysis and RNA interference (*RNAi*) studies demonstrate that activation of the AdpR is associated with an inhibition of mosquito *lipophorin* expression. Interestingly, lipophorin-mediated pathways enhance *Plasmodium* survival in mosquitoes (17), and *AdpR*-deficient mosquitoes are more readily colonized by *Plasmodium*. This study identifies a mosquito pathway triggered by a component of blood meal, adiponectin, which negatively impacts *Plasmodium* infection in its vector, mosquitoes. This is an example of cross-talk between the mosquito and the vertebrate host on which it feeds, which is detrimental to *Plasmodium* infection in the arthropod.

## RESULTS

### Characterization of an *Anopheles* adiponectin receptor protein

**Fig 1:**
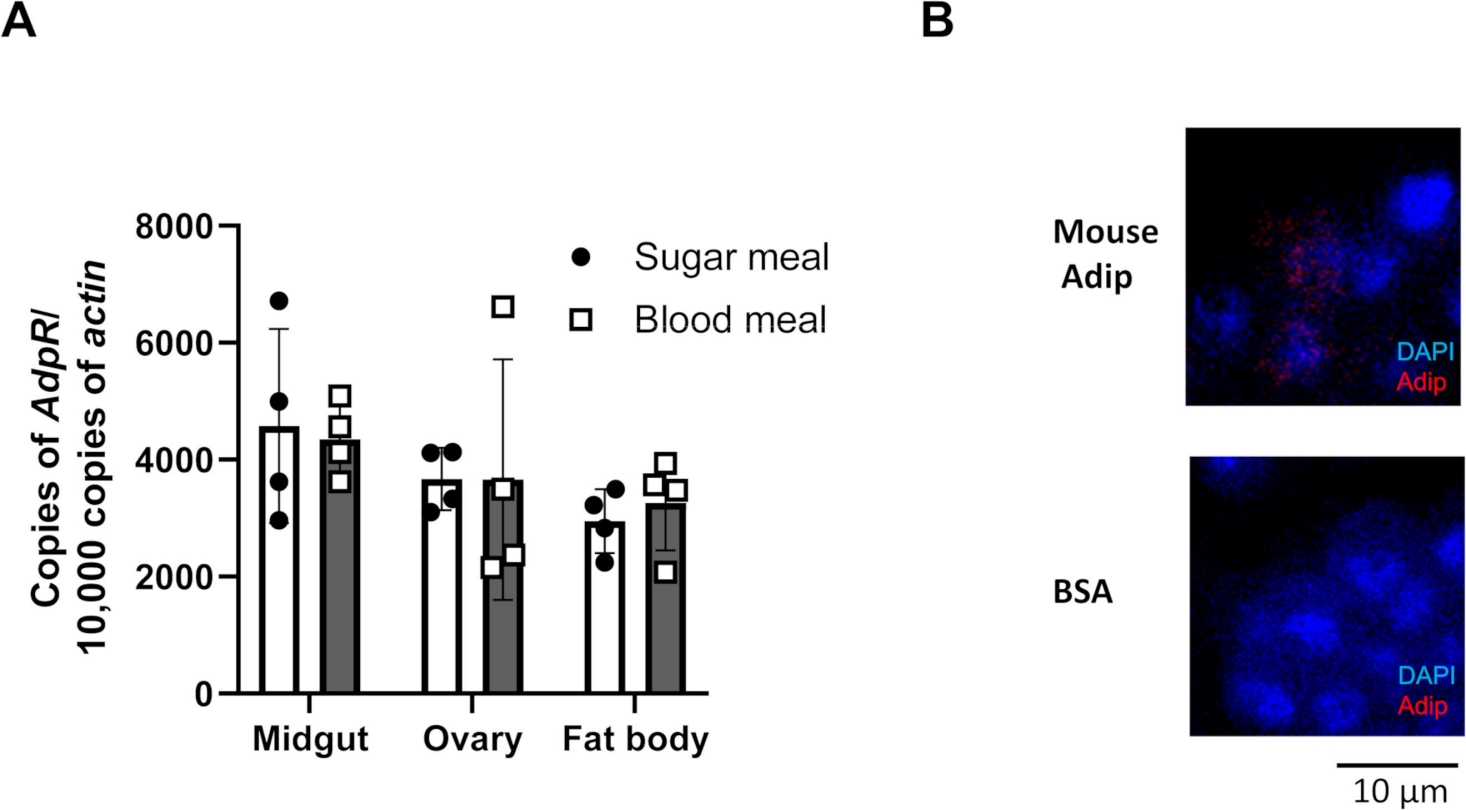
Mammalian adiponectin co-localizes with the mosquito midgut. (A) Following a sugar meal or blood meal, mRNA from the mosquito midgut, ovary, or fat body was collected. The expression level of the adiponectin receptor was determined by RT-PCR. Each dot in the midgut and ovary groups represents a pooled sample from two mosquitoes. Each dot in the fat body group represents a pooled sample from four mosquitoes. (Mean ± S.D.) (B) Murine adiponectin bound to mosquito midguts. His-tag murine adiponectin or control protein (BSA) was incubated with the mosquito midgut, and the presence of adiponectin in the midgut was determined by confocal microscopy.

When mosquitoes take a blood meal, components of mammalian blood interact with the arthropod (26–29). These relationships have the potential to influence metabolic pathways in the mosquito and modulate *Plasmodium* infection in its vector (30, 31). A systematic gene search demonstrates that *AGAP004486* encodes a putative adiponectin receptor (AdpR) in *A. gambiae,* which has substantial homology with the AdpRs in humans, mice, and rats (Table S1). The full-length *AdpR* encodes a protein with 425 amino acid residues and 65% amino acid sequence similarity to both human and murine AdpR 1 and 2. Compared with human and murine AdpR 1 and 2 (32), the homologous seven transmembrane domains and zinc-binding sites, the 3× His and Asp residues and a Ser residue, are well-conserved between mosquito AdpR and mammalian AdpRs (Table S1). In contrast, the *A. gambiae* genome does not contain a gene with homology to adiponectin. To evaluate the expression levels of *AdpR* in different tissues, we collected midguts, ovaries, and fat bodies from mosquitoes after feeding. By reverse transcription quantitative polymerase chain reaction (RT-qPCR), the expression level of *AdpR* in the midgut, ovaries, and fat bodies did not show any statistically significant differences (Fig. 1A). In addition, there was no significant change after a blood meal (Fig. 1A). As *A. gambiae* has a putative AdpR but lacks adiponectin, we determined whether mammalian adiponectin can be identified on the midgut surface. The midgut was collected and incubated with murine adiponectin. Recombinant murine adiponectin could be detected on the surface of some midgut cells, compared with controls (Fig. 1B).

### *Plasmodium berghei* infection of mosquitoes is altered by the lack of adiponectin

**Fig 2:**
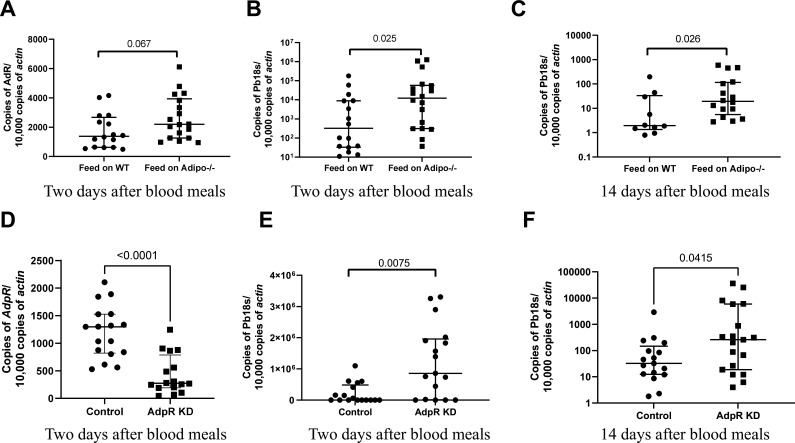
Lack of adiponectin in the incoming blood meal increases infection of *Anopheles gambiae* with *Plasmodium berghei*. (A) *P. berghei*-infected RBCs were collected from *Adipoq^-/-^* mice, and then equal amounts of plasma from *Adipoq^-/-^* mice or wild-type mice were mixed with the infected RBCs. Mosquitoes were fed on blood lacking adiponectin or wild-type plasma by membrane feeding. Two days after the blood meal, the midguts were collected, and the expression levels of the adiponectin receptor (**A**) and the burden of *Plasmodium* were determined by RT-qPCR (**B**). (C) Fourteen days after the blood meal, the midguts of mosquitoes were collected and the burden of *Plasmodium* was determined by RT-qPCR. (D) Mosquitoes were injected with *dsAdpR* (*AdpR* knockdown, AdpR KD), or *dsGluc* (control). Two days after ds*RNA* injection, the mosquitoes were fed on *P. berghei*-infected mice. Two days after the blood meal, the midguts were collected and the expression levels of adiponectin receptor (**D**) and the burden of *Plasmodium* were determined by RT-PCR (**E**). (F) Fourteen days after the blood meal, the midguts of mosquitoes were collected, and the burden of *Plasmodium* was determined by RT-PCR. Each dot represents one mosquito. (Median ± interquartile range, *P* < 0.05 using the Mann-Whitney U-test.)

As *Plasmodium* are acquired by mosquitoes from the vertebrate host during a blood meal, we postulated that interactions between mosquito AdpR and mammalian blood could potentially affect *Plasmodium* infection of mosquitoes. We collected *Plasmodium*-infected red blood cells (RBCs) from *adiponectin*-knockout (KO, *Adipoq^-/-^*) mice and then mixed the cells with plasma from wild-type or *adiponectin*-KO infected blood. Mosquitoes were then fed on this blood by membrane feeding. The expression of *AdpR* in the midgut was similar, regardless of the presence of adiponectin in the blood meal (Fig. 2A). There was a significantly higher *P. berghei* burden in the mosquito midgut, 2 and 14 days after taking the blood meal with plasma lacking adiponectin (Fig. 2B and C, *P* < 0.05). This suggests that adiponectin indirectly impedes *Plasmodium* infection of mosquitoes.

### Silencing mosquito *AdpR* enhances *P. berghei* infection

Since mammalian adiponectin may interact with the *A. gambiae,* AdpR and the absence of adiponectin in a blood meal enhanced *Plasmodium* infection of mosquitoes, we hypothesized that *RNAi-*mediated silencing of the *A. gambiae AdpR* would increase *Plasmodium* transmission to mosquitoes. To silence *AdpR*, *AdpR* or *Gaussia luciferase* (*Glu*, control) dsRNA was injected into the thorax of naïve mosquitoes. Two days later, the mosquitoes were allowed to feed on the same *P. berghei*-infected mouse. RT-qPCR showed a significant decrease of *AdpR* expression in the midguts of ds*AdpR*-injected mosquitoes when compared to that in the midguts of control ds*Gluc*-injected mosquitoes (Fig. 2D, *P* < 0.001), indicating that the expression of *AdpR* was successfully decreased. Two or 14 days after feeding, *AdpR*-silenced mosquitoes showed a marked increase in the *P. berghei* burden in the midguts when compared to that in control mosquitoes (Fig. 2E and F, *P* < 0.05). To further demonstrate that an interaction between mammal adiponectin and mosquito AdpR affects the transmission of *Plasmodium* to mosquitoes, we let control (dsGluc) and *AdpR*-silenced (dsAdpR) mosquitoes fed on *P. berghei-*infected adiponectin-knockout (*Adipoq^-/-^)* mice. There was no difference in the *Plasmodium* burden 2 days after blood meal (Fig. S1A). These data suggest that an association between murine adiponectin and the mosquito adiponectin receptor alters the establishment of *Plasmodium* infection in mosquitoes.

### AdpR-related pathways associated with *P. berghei* infection of mosquitoes

**Fig 3:**
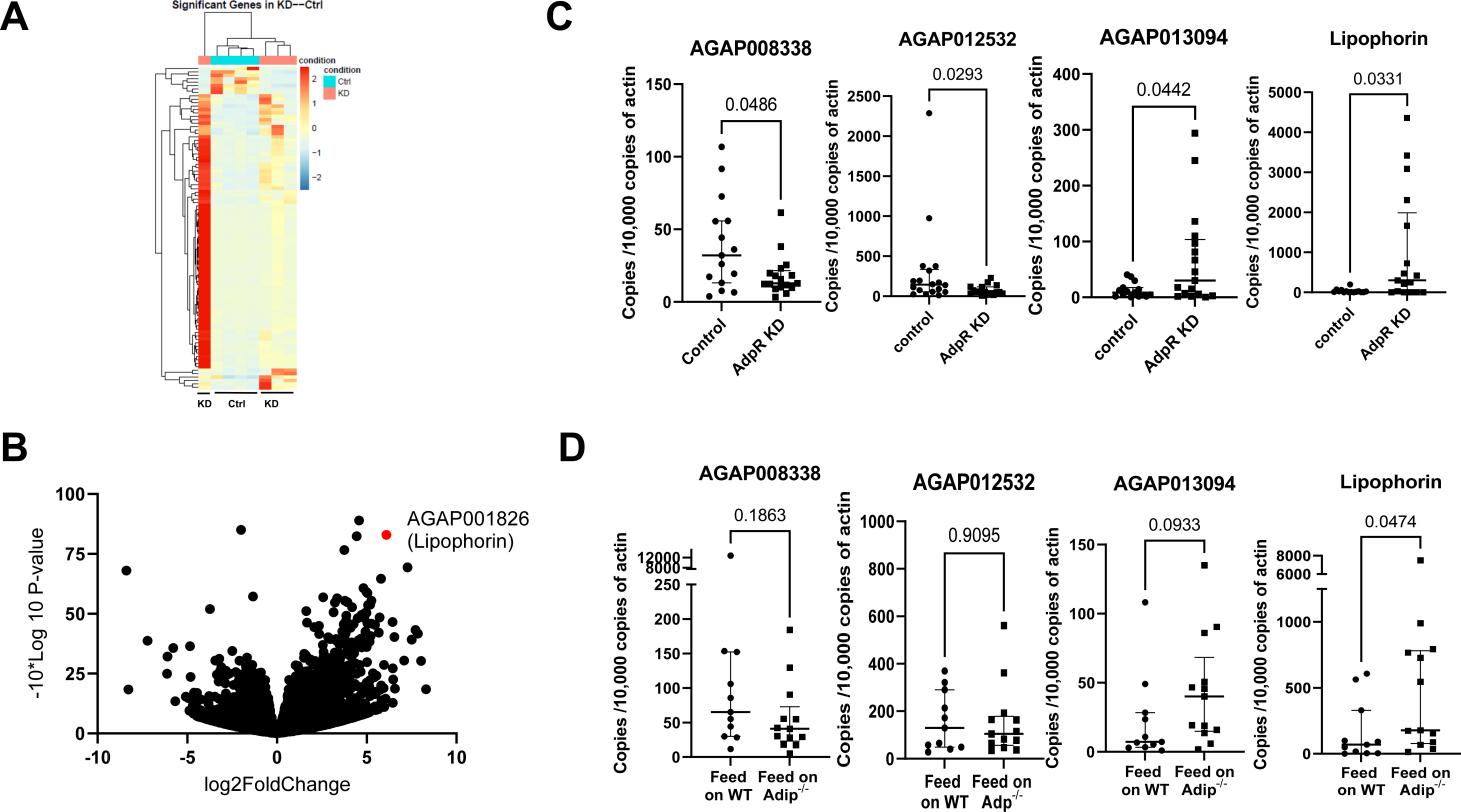
Gene expression analysis reveals that lipophorin (AGAP001826) is regulated by the adiponectin receptor. Mosquitoes were injected with *dsAdpR* (*AdpR* knockdown, KD) or *dsGluc* (control, Ctrl). Two days after ds*RNA* injection, the mosquitoes were fed on *P. berghei*-infected mice. Two days after the blood meal, each midgut was collected, and RNA from each midgut was isolated for RNA-seq analysis. (A) A hierarchical clustering of differentially expressed genes was generated by comparing data from *AdpR* knockdown and control mosquitoes. (B) Volcano plots showing significant changes in gene expression between *AdpR* knockdown and control mosquitoes. Dispersion graph of –10*log10(*P* value) (y-axis) against the log2(fold change) (x-axis) corresponding to the genes by their differential expression. (C) RT-qPCR validation of gene expression in the mosquito midgut after injection with ds*AdpR* (Adiponectin knockdown, AdpR KD) or ds*Gluc* (control). Two days after ds*RNA* injection, mosquitoes were fed on *P. berghei*-infected mice. Two days after the blood meal, the midguts were collected for RT-qPCR. (D) RT-qPCR validation of expression in mosquito midguts 2 days after feeding on adiponectin knockout (Adipoq^-/-^) or wild-type *P. berghei*-infected mice. Each dot represents one mosquito. (Median ± interquartile range, *P* < 0.05 using the Mann-Whitney U-test.)

To investigate the mechanisms underlying the association of *AdpR* and *P. berghei* infection, we assessed the presence or absence of *AdpR* on mosquito physiology by comparing transcriptomes of *AdpR-silenced* and control *A. gambiae* 2 days after feeding on *P. berghei*-infected mice. After taking a blood meal on *P. berghei*-infected mice, 94 genes were significantly differentially expressed with 86 upregulated and 8 downregulated genes in the midguts of *AdpR*-silenced mosquitoes when compared to that in control mosquitoes (Fig. 3A and B; Table S2). The transcriptome analysis also demonstrated that the *AdpR* gene was successfully silenced by *RNAi*, consistent with our RT-qPCR results. One of the most significantly upregulated genes was *lipophorin* (adjusted *P* value < 10^−4^), which has been reported to play an important role in lipid transport and mosquito egg development (15, 16). In addition, several lipid homeostasis-associated genes, including *fatty acyl-CoA reductase 2* (*AGAP005985*) or *elongation of very long chain fatty acids protein* (*AGAP013094*), were also upregulated in the absence of *AdpR* after feeding on *P. berghei-*infected mice, further suggesting that AdpR may have a role in mosquito metabolism after a blood meal. Transcriptomic data also demonstrated that *AdpR* silencing resulted in the upregulation of CLIP domain serine proteases (Table S1).

### The interaction between mammalian adiponectin and *A. gambiae* AdpR mediates the expression of lipophorin

To further investigate the downstream effects of the interaction of mammalian adiponectin and mosquito AdpR, we selected three downregulated and six upregulated genes to validate the accuracy and reproducibility of the transcriptome bioinformatic analyses by RT-qPCR (Fig. 3C; Fig. S1). The RT-qPCR samples were collected from the midguts of *AdpR*-silenced or control mosquitoes, which were not part of the initial sequencing samples, 2 days after a blood meal. The RT-qPCR results indicate that *AGAP008338* and *AGAP012532* were significantly downregulated after silencing of *AdpR,* and that *lipophorin* and *AGAP013092* were significantly upregulated (Fig. 3C, *P* < 0.05). At the same time, the difference between the other four genes has a similar trend as the RNAseq data, but without statistical significance (Fig. S2). Next, we determined whether the expression of these four genes was affected when mosquitoes fed on a blood meal that lacked adiponectin. Among the four genes, *lipophorin* was also significantly upregulated 2 days after mosquitoes fed on *P. berghei*-infected adiponectin-knockout mice (Fig. 3D, *P* < 0.05). Based on these transcriptomic analyses, lipophorin is negatively regulated by signals associated with the adiponectin receptor and mouse adiponectin.

**Fig 4:**
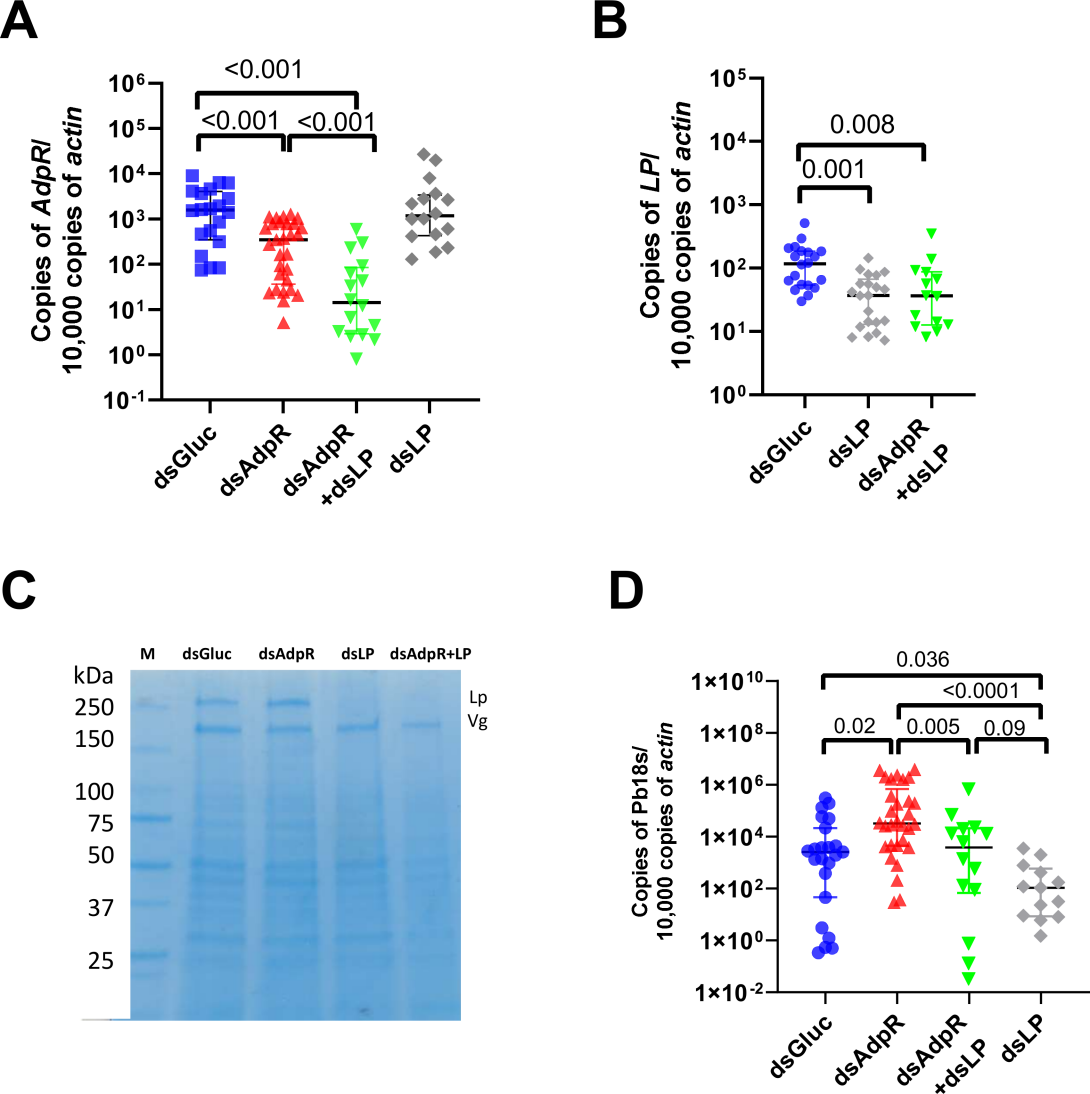
Adiponectin signaling affects *Plasmodium* infection in mosquitoes through the lipophorin pathway. Mosquitoes were injected with ds*AdpR* (*AdpR* knockdown), ds*Lp* (*lipophorin* knockdown), both ds*AdpR* and ds*Lp,* or *dsGluc* (control). Two days after injection, the mosquitoes were fed on *P. berghei*-infected mice. Two days after the blood meal, the midguts were collected, and the expression levels of *adiponectin receptor* (**A**) and *lipophorin* (**B**) were determined by RT-qPCR. (C) Hemolymph from 10 knockdown or control mosquitoes was collected, and the levels of lipophorin (Lp) and vitellogenin (Vg) were determined by SDS-PAGE gel. (D) The *Plasmodium* burden was determined by RT-qPCR. Each dot represents one mosquito. (Median ± interquartile range, *P* < 0.05 using the Mann-Whitney U-test.)

Since *Plasmodium* infection can be enhanced by lipophorin (13, 15) and lipophorin is negatively regulated by adiponectin receptor, we next determined whether the enhancement of *Plasmodium* infection in mosquitoes due to silencing *AdpR* was mediated by *lipophorin (Lp)*. We silenced *AdpR* and *Lp* together to investigate their effects on *P. berghei* infection. By RT-qPCR, we confirmed that co-injection of ds*AdpR* and ds*Lp* significantly decreased the expression level of both *AdpR* and *Lp* (Fig. 4A and B). Silencing of *Lp* did not affect the expression of the adiponectin receptor (Fig. 4A), which indicated that it is unlikely that *AdpR* functions downstream of *Lp*. The decreased synthesis of lipophorin protein in ds*Lp* group was also confirmed by SDS-PAGE (Fig. 4C). After feeding on *P. berghei*-infected mice, mosquitoes that had both *AdpR* and *lipophorin* silenced had a similar *Plasmodium* burden as the control group and a significantly lower *Plasmodium* infection level compared to the *AdpR*-silenced group (Fig. 4D). When *Lp* was silenced, the *Plasmodium* infection level was significantly decreased compared to the control (dsGluc) and *AdpR* silencing group (Fig. 4D). There was no difference between the ds*Lp*, and the *AdpR* and *Lp* silencing groups (Fig. 4D). Based on our studies, the effects of silencing *AdpR* on *Plasmodium* infection in mosquitoes depend on the lipophorin pathway.

### *AdpR* affects *Plasmodium* development in the mosquito midgut

**Fig 5:**
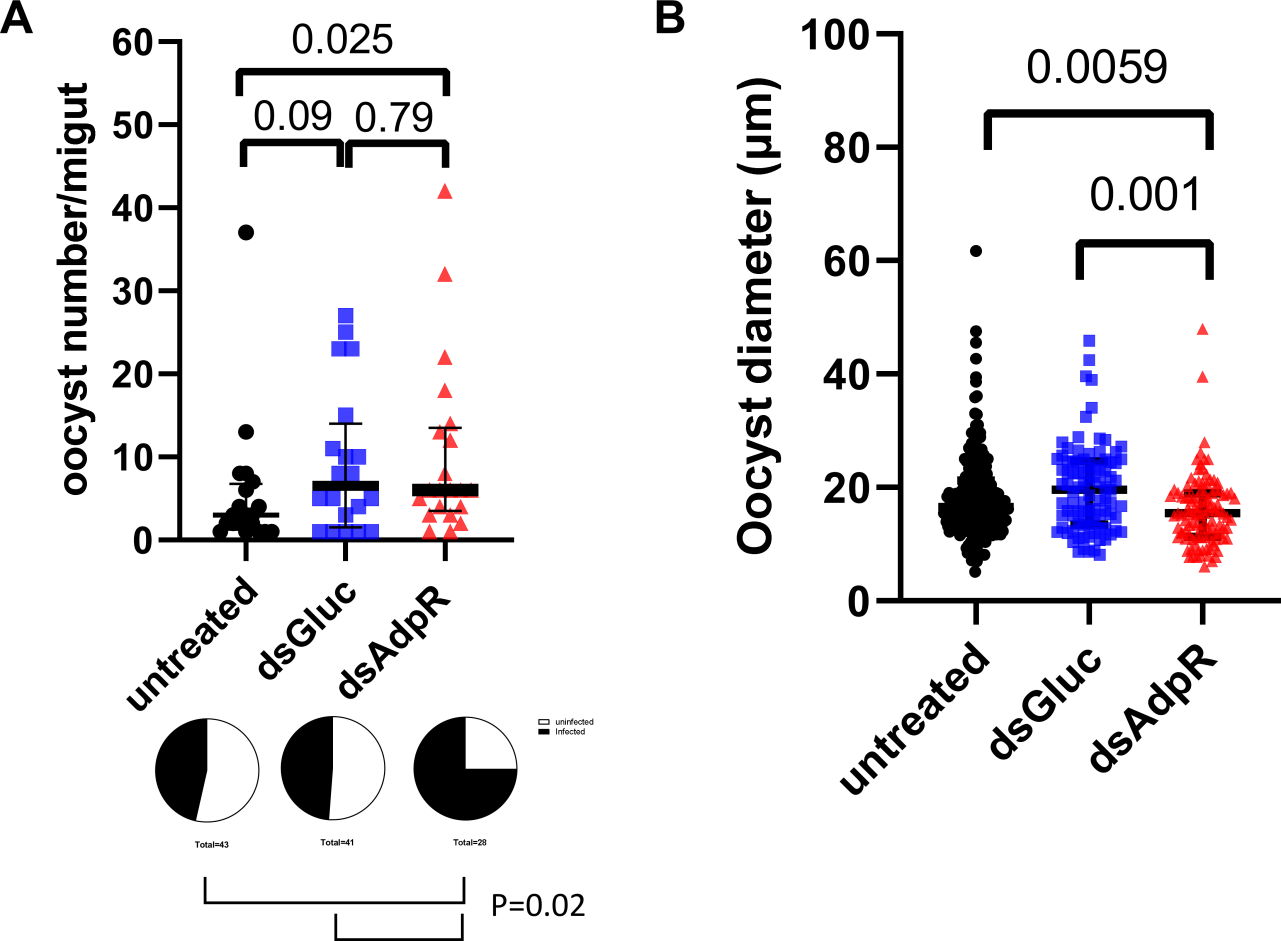
Silencing of adiponectin signaling affects the oocyst number and size in mosquitoes. Mosquitoes were injected with ds*AdpR* (*AdpR* knockdown) or *dsGluc* (control). Two days after injection, the mosquitoes were fed on GFP-expressing *P. berghei*-infected mice. (A) Seven days after the blood meal, the midguts were collected. The number of oocysts per midgut was determined by the GFP signals using EVOS fluorescence microscopy. The dot blots represent the number of oocysts from each group of infected midguts. (Median ± interquartile range (IQR), *P* < 0.05 using the Mann-Whitney U-test.) The pie charts represent the percentage of infected mosquitoes in each group (*P* < 0.05 using the Chi-square test). (B) The size of oocysts in each group was determined by GFP signals using EVOS fluorescence microscopy. (Median ± IQR, *P* < 0.05 using the Mann-Whitney U-test.)

To further understand the impact of the adiponectin receptor on the development of *Plasmodium* in the midgut, we examined the number of oocysts in the midguts in *AdpR-*silenced mosquitoes. Seven days after taking a blood meal from GFP-expressing *P. berghei*-infected mice, the midguts from control mosquitoes (both untreated and ds*Gluc-*treated) and *AdpR*-silenced mosquitoes were collected to determine the number and size of oocysts. By counting the oocysts, there was a significant higher prevalence of infection in *AdpR*-silenced mosquitoes (75%) compared to control (46.5%) or ds*Gluc-*treated mosquitoes (48.8%) (*P* = 0.02 by the Chi-square test, Fig. 5A). There was a significant increase in the oocyst number in *AdpR*-silenced mosquitoes compared to untreated mosquitoes (Fig. 5A, *P* < 0.05). There was no difference between untreated and ds*Gluc*-treated or ds*Gluc*-treated and *AdpR*-silenced mosquitoes. In contrast to the increased number of oocysts, the diameters of oocysts were slightly smaller in *AdpR*-silenced mosquitoes compared to untreated mosquitoes or the ds*Gluc-treated* mosquitoes (Fig. 5B, *P* < 0.05). Furthermore, we let control (dsGluc) and *AdpR*-silenced (dsAdpR) mosquitoes fed on *P. berghei-*infected adiponectin-knockout (*Adipoq^-/-^)* mice. There was no difference in the prevalence and oocyst number between these two groups when there was a lack of adiponectin in the blood meal (Fig. S1B). All these results demonstrate that silencing of adiponectin receptor affects the development of oocysts in the midgut.

## DISCUSSION

Hematophagous arthropods acquire blood from other animals to obtain nutrients for physiological and reproductive needs. Nutrients in the blood directly influence fitness (26). However, other factors in blood can indirectly influence both fitness and *Plasmodium* survival (3). A human miRNA, hsa-miR-150–5p, released from blood, can be disseminated into the mosquito hemocoel and suppress antiviral genes, an example of cross-species RNAi-mediated gene regulation (33). Insulin in a blood meal can bind to an insect insulin-like peptide receptor in mosquitoes and tsetse flies (34–36). In *Anopheles* mosquitoes, administration of insulin can increase the *Plasmodium* burden (35–37). All these results demonstrate the intimate interaction between the vertebrate host, *Plasmodium,* and mosquitoes.

Female mosquitoes modulate lipid metabolism after a blood meal to complete vitellogenesis . Following engorgement, the secretion of 20E leads to an upregulation of lipophorin and vitellogenin, a prerequisite for egg development in the ovary. Lipophorin is important for egg maturation, and *Plasmodium* utilize mosquito lipids through lipophorin (13, 15, 17). Silencing lipophorin led to fewer ookinetes and oocysts in the midgut of mosquitoes (13, 17). In our study, we demonstrate that silencing the adiponectin receptor led to upregulation of lipophorin and that there were increased number of oocysts in the *AdipR*-silenced mosquitoes, which is consistent with the impact of lipophorin on oocyst development (9, 13). The oocysts were slightly smaller in size in the *AdipR*-silenced mosquitoes, while oocyst size usually increases when lipophorin is upregulated (9, 13). This suggests that other factors may influence oocyst size. Further studies will elucidate the diverse factors related to how *AdpR* interactions alter various aspects of oocyst development.

The transcriptomic data also demonstrated that *AdpR* silencing resulted in the modest upregulation of CLIP domain serine proteases (Table S2). Silencing of *CLIPA9* in mosquitoes leads to a lower *P. falciparum* oocyst intensity in the midguts of mosquitoes (38), and knockdown of either *CLIPA2/CLIPA5* induces mosquito melanization and directly kills ookinetes (39). Taking together, the increased expression of CLIP domain serine proteases following *AdpR* silencing may also contribute to alterations in *Plasmodium* infection. Further studies will be needed to clarify the impact of CLIP domain serine proteases on *Plasmodium* development after *AdpR* silencing.

In contrast to the role of the adiponectin receptor during *Plasmodium* infection in mosquitoes, ISARL, an adiponectin-like receptor in ticks also serves as a metabolic regulator, but it is activated by a C1q-like protein in ticks and not by mammalian adiponectin (25). When ISARL is silenced in ticks, a critical phospholipid metabolism enzyme, PTDSS1, is affected (25). Silencing of ISARL significantly reduced the *Borrelia burgdorferi* burden in the tick (25). Unlike ISARL, we found that silencing of mosquito AdpR and the absence of adiponectin in the murine blood meal led to a similar effect on the acquisition of *Plasmodium.* There was also a similar effect on the expression of lipophorin, all of which suggest that the mosquito adiponectin receptor may be modulated by an incoming blood meal which contains mammalian adiponectin. Since different species of insects have homologues of adiponectin receptor proteins (40), it is possible that other ligands, such as C1q-like proteins, can activate the adiponectin receptor. While homology searches do not uncover any C1q-like protein in mosquitoes, we cannot exclude the possibility that there are additional unknown ligands for the adiponectin receptor in mosquitoes. In contrast to the positive effect of ISARL on *Borrelia burgdorferi* burden (25), there is a negative effect on the *Plasmodium* burden after activation of the mosquito adiponectin receptor. Therefore, different downstream effects in various arthropods can lead to the distinct effects in specific vector-borne pathogens.

In summary, we identified a novel pathway involving an *A. gambiae* adiponectin receptor, which triggered by adiponectin from an incoming blood meal, decreases *Plasmodium* infection in the mosquito. Activation of this pathway negatively regulates lipophorin expression, an important lipid transporter that both enhances egg development and *Plasmodium* infection. The relative cost involved in altering egg development and *Plasmodium* infection may depend on the overall fitness of the mosquito. This is an unrecognized cross-phyla interaction between a mosquito and a mammal on which it feeds. These processes are critical to understanding the complex life cycle of mosquitoes and *Plasmodium* following a blood meal and may be applicable to other hematophagous arthropods and vector-borne infectious agents.

## MATERIALS AND METHODS

### Animals

*A. gambiae* (4arr strain) mosquitoes were raised at 27°C, 80% humidity, under a 12/12-hour light/dark cycle and maintained with 10% sucrose under standard laboratory conditions in the insectary at Yale University. Swiss Webster mice were purchased from Charles River Laboratories. C57BL/6 J mice wild type (WT) and C57BL/6 J mice deficient in adiponectin (*Adipoq^-/-^)* were purchased from the Jackson Laboratory.

### Hemolymph collection

Hemolymph was collected as previously described (41, 42). Briefly, mosquitoes were placed on ice and the proboscis was clipped. The lateral aspects of the thorax were gently pressed using fine forceps to push out a drop of hemolymph from the cut site. Hemolymph droplets from 10 *dsRNA* knockdown or control mosquitoes were collected into Laemmli sample buffer using a pipette tip. Proteins in the hemolymph were separated by SDS-PAGE and analyzed by Coomassie staining.

### *dsRNA* synthesis and microinjection

The *adiponectin receptor* gene (*AGAP004486*) fragment between base pairs 37 and 564 of the coding sequence and the *lipophorin* gene (*AGAP001826*) fragment between base pairs 9370 and 9936 of the coding sequence were amplified from cDNA collected from midguts using PCR. *Adiponectin receptor* (*AdpR*) and *lipophorin* dsRNAs were further synthesized from the PCR-amplified fragments using a T7 RiboMAX kit (Promega). The primer sequences are listed in Table S3 in the supplemental material. *Gaussia luciferase* dsRNA was used as the control for dsRNA injection. Two days before the blood meal, 100 ng of dsRNA was injected into the thoraces of female mosquitoes under ice anesthesia. The injection was performed using a nanoinjector (Nanoject II; Drummond) with a glass capillary needle, as previously described (43).

### Gene expression and *Plasmodium* load

The RNeasy Mini Kit (QIAGEN, CA) was used to extract RNA from the midguts, ovaries, and fat bodies of the mosquitoes. All extractions followed the manufacturer’s protocols. The iScript RT-qPCR kit (Bio-Rad, CA) was used to generate cDNA from RNA. Using iTaq SYBR Green Supermix (Bio-Rad, CA), real-time PCR was performed on a CFX96 real-time platform (Bio-Rad). PCR involved an initial denaturation at 95°C for 2 min, 50 cycles of 15 sec at 95°C, 15 sec at 60°C, and 20 sec at 72°C. Fluorescence readings were taken at 72°C after each cycle. At the end of each reaction, a melting curve (60–95°C) was checked to confirm the identity of the PCR product. The burden of *Plasmodium* in midguts after blood meals was determined by assessing the expression level of *P. berghei 18S rRNA,* normalized to *A. gambiae actin mRNA.* These data were presented as copy number of the target gene per 10,000 copies of the housekeeping gene, *actin*. The primers used for the expression of sporozoite genes are listed in Table S3.

### Transcriptome analyses

dsRNA (ds*AdpR* and ds*Gluc*) microinjected *A. gambiae* mosquitoes were fed on *P. berghei*-infected Swiss Webster mice. Two days after a blood meal, the mosquitoes were collected for midgut dissection. Total RNA was purified as described above. RNA samples were then submitted for library preparation using TruSeq (Illumina, San Diego, CA, USA) and sequenced using Illumina HiSeq 2500 by paired-end sequencing at the Yale Centre for Genome Analysis (YCGA). The transcript data were mapped with the *A. gambiae* PEST genome (AgamP4.13) downloaded from VectorBase (https://vectorbase.org/vectorbase/app/) (44) and indexed using the kallisto-index (45). The reads from the sequencer were pseudo-aligned with the index reference transcriptome using kallisto (45). The counts generated were processed by DESeq2 (46) in RStudio (https://rstudio.com). The transcriptomic data are available in the Gene Expression Omnibus repository at the National Center for Biotechnology Information under the accession number: GSE198929.

### Binding assays of murine adiponectin in mosquito midgut

The midgut was dissected from the mosquitoes and then incubated with 1 µg/µL of mouse adiponectin (SinoBiological, #50636-M08H), or BSA for 1 hour. The tissues were washed with PBS and fixed in 4% PFA for 15  min at room temperature. Then, the cells were blocked in 1% BSA in PBS for 1 hour, and subsequently immunolabeled with an Alexa Fluor 555 anti-His antibody (1:100, ThermoFisher, # MA1-21315-A555) for 1 hour. Nuclei were stained with DAPI (Invitrogen, #D9542). After staining, the fluorescence signals were examined with a Leica SP5 confocal microscope.

### *P. berghei* infection

*P. berghei* (NK65 RedStar or ANKA GFP, ATCC) were maintained by serial passage in 6 to 8-week-old female Swiss Webster or C57BL/6 mice as previously described (47). Briefly, Swiss Webster or C57BL/6 mice were challenged with *P. berghei*-infected RBCs by intraperitoneal injection. *A. gambiae* mosquitoes then took a blood meal from the infected mice, when the parasitemia was approximately 5%. Seventeen to 24 days after *P. berghei* infection, the mosquitoes were sorted using the fluorescent signal of the salivary glands.

For artificial membrane feeding, mosquitoes were starved for at least 17 hours before exposure to blood. Blood was collected from *Adipoq^-/-^* or wild-type mice infected with *P. berghei* and treated with EDTA to prevent coagulation. Blood samples were centrifuged at 2,000 *g* for 5 min. The RBCs from *Adipoq^-/-^* mice were mixed with an equal amount of plasma from *Adipoq^-/-^* or wild-type mice. The blood was then transferred into a Hemotek blood reservoir unit. Mosquitoes were then fed at a constant temperature of 37°C using the Hemotek blood feeding system with stretched Parafilm as a membrane. After 30 min of blood feeding, mosquitoes were kept in paper cups covered with net and maintained as described above.

### Oocyst numbers and sizes

Seven days post-feeding on *P. berghei* (ANKA GFP) infected mice, the midguts of mosquitoes that had completed a blood meal were dissected, and the midguts were evaluated with a EVOS fluorescence microscope. The number and size of oocysts in the midguts were determined using GFP as a marker (48).

### Statistical analysis

All experiments were repeated at least three times. Data from at least three biological replicates were used to calculate medians for graphing purposes. Statistical analyses employed the Mann-Whitney test to determine the difference, and the data were presented as the median with interquartile range (IQR) or mean with standard deviation (SD). A *P-*value of <0.05 was considered statistically significant. The analysis, graphs, and statistics of all data were performed using Prism 9.0 software (GraphPad Software).

## Data Availability

The transcriptomic data are available in the Gene Expression Omnibus (GEO) repository at the National Center for Biotechnology Information under the accession number: GSE198929.

## SUPPLEMENTAL MATERIAL

Fig. S1 mbio.02257-23-s0001.tif Lacking adiponectin in the incoming blood meal abolishes the effects of the adiponectin receptor on the infection of *Plasmodium berghei* in *A. gambiae*.

Fig. S2 mbio.02257-23-s0002.tif Gene expression analysis by RT-qPCR when silencing *AdpR*.

Legends mbio.02257-23-s0003.docx Supplemental figure legends.

Table S1 mbio.02257-23-s0004.docx Comparison of adiponectin receptors by protein sequences.

Table S2 mbio.02257-23-s0005.docx *Anopheles gambiae* genes that are significantly changed (adjusted *P* < 0.05) in mosquitoes which the adiponectin receptor gene has been silenced, compared with control mosquitoes.

Table S3 mbio.02257-23-s0006.docx Primers used in the studies.

ASM does not own the copyrights to Supplemental Material that may be linked to, or accessed through, an article. The authors have granted ASM a non-exclusive, world-wide license to publish the Supplemental Material files. Please contact the corresponding author directly for reuse.
