# Supplementary figures and images for: Signaling between mammalian adiponectin and a mosquito adiponectin receptor reduces Plasmodium transmission
Source: mBio. 2023 Dec 11;15(1):e02257-23. doi: 10.1128/mbio.02257-23 (PMC10790699; doi:10.1128/mbio.02257-23)

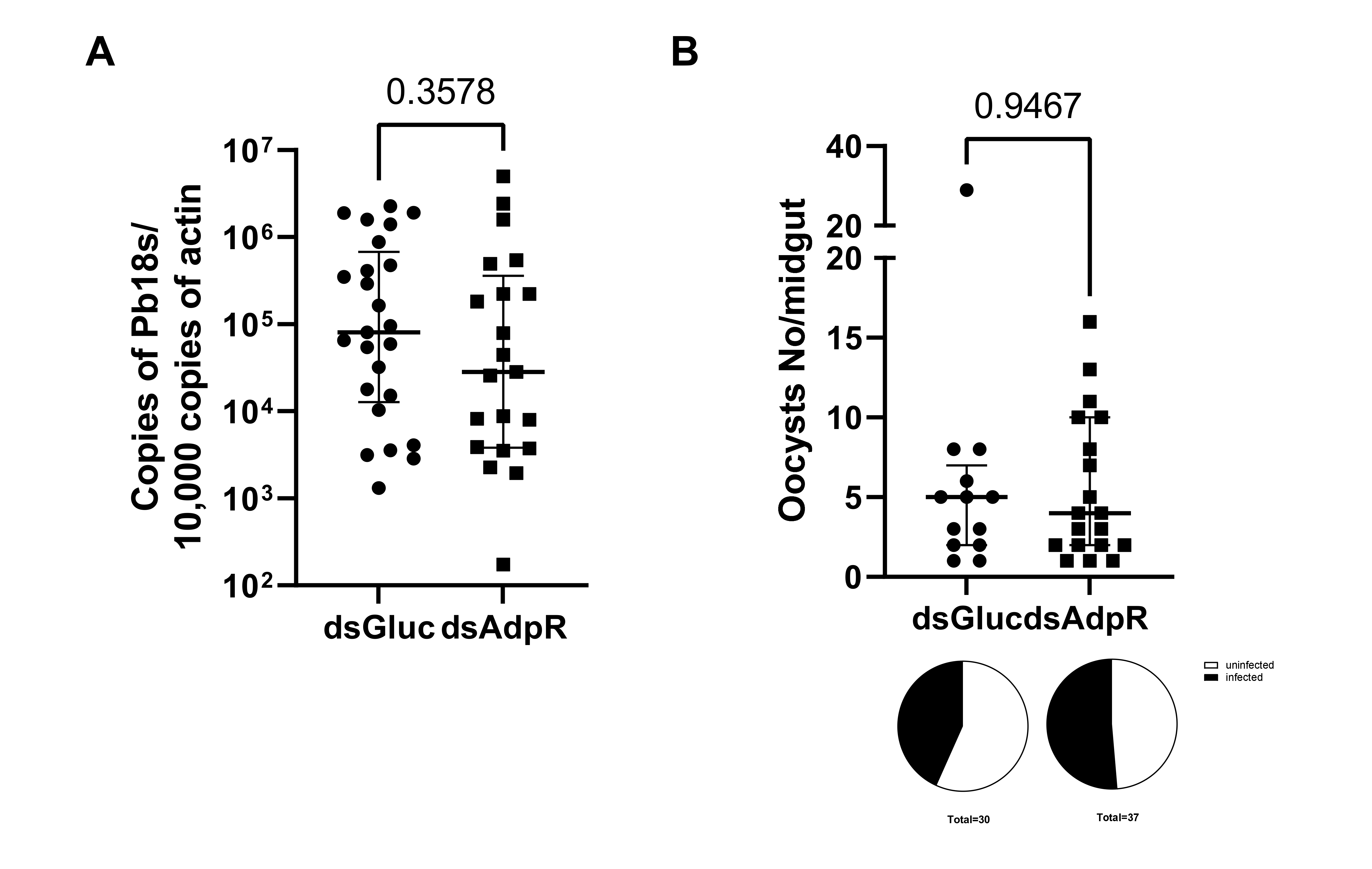

Supplement: Fig. S1 — Lacking adiponectin in the incoming blood meal abolishes the effects of the adiponectin receptor on the infection of Plasmodium berghei in A. gambiae. [file mbio.02257-23-s0001.tif]

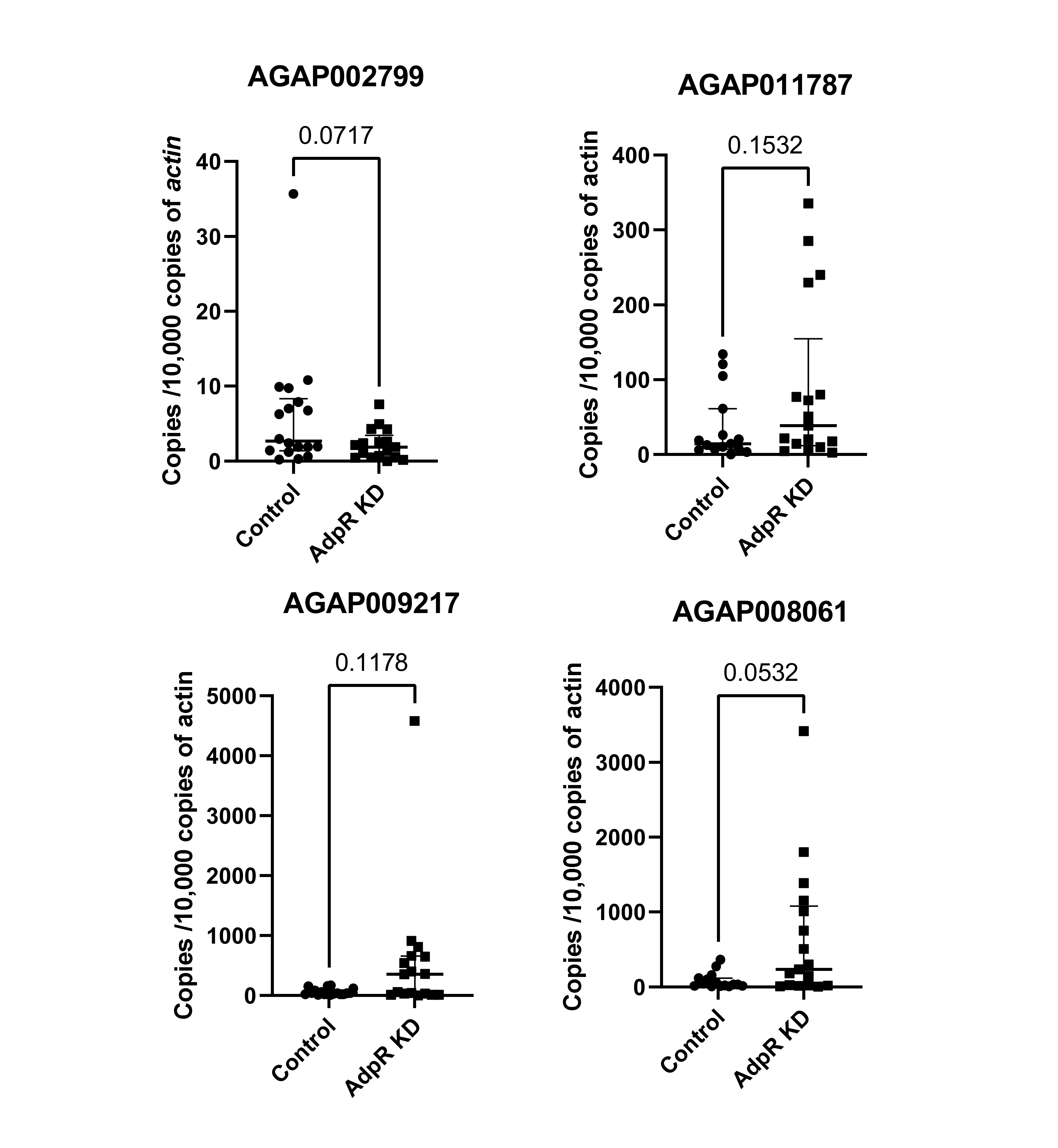

Supplement: Fig. S2 — Gene expression analysis by RT-qPCR when silencing AdpR. [file mbio.02257-23-s0002.tif]
