# Supplementary material for: Signaling between mammalian adiponectin and a mosquito adiponectin receptor reduces Plasmodium transmission
Source: mBio. 2023 Dec 11;15(1):e02257-23. doi: 10.1128/mbio.02257-23 (PMC10790699; doi:10.1128/mbio.02257-23)
Supplement: Legends — Supplemental figure legends. [file mbio.02257-23-s0003.docx]

**Supplement Figure Legends**

**Figure S1. Lacking adiponectin in the incoming blood meal abolishes the effects of the adiponectin receptor on the infection of *Plasmodium berghei in A. gambiae*.**  A. Mosquitoes were injected with *dsAdpR* (*AdpR* knockdown, AdpR KD) or *dsGluc* (control). 2 days after injection, the mosquitoes were fed on the same *P. berghei*-infected *Adipoq^-/-^* mouse. Two days after the blood meal, the midguts were collected and the burden of *Plasmodium* was determined by RT-PCR. B. Seven days after the blood meal, the midguts of mosquitoes were collected and the oocyst numbers determined by GFP signal. Each dot represents the number of oocysts from one infected mosquito. (Median ± IQR, *p value* by the Mann Whitney U-test). The pie charts represent the percentage of infected mosquitoes in each group.

**Figure S2. Gene expression analysis by RT-qPCR when silencing *AdpR.*** To validate the genes expression result from RNA-seq, mosquitoes were injected with dsAdpR (Adiponectin knockdown, AdpR KD) or dsGluc (control). Two days later, mosquitoes were fed on *Plasmodium berghei*-infected mice. The midguts were collected for RT-qPCR to analysis the expression level of *AGAP002799, AGAP011787, AGAP009217* and *AGAP008061*. Each dot represents one mosquito. (Median ± IQR, p<0.05 using Mann Whitney U-test)
