# Supplementary material for: Signaling between mammalian adiponectin and a mosquito adiponectin receptor reduces Plasmodium transmission
Source: mBio. 2023 Dec 11;15(1):e02257-23. doi: 10.1128/mbio.02257-23 (PMC10790699; doi:10.1128/mbio.02257-23)
Supplement: Table S1 — Comparison of adiponectin receptors by protein sequences. [file mbio.02257-23-s0004.docx]

**Table S1. Comparison of adiponectin receptors by protein sequences.**

Multiple sequence alignment of *Anopheles gambiae* adiponectin receptor (AGAP004486, A gambAdpR) with the amino acid sequences of homologs identified in *Homo sapiens* (Q96A54, HumanAdpR1; Q86V24, HumanAdpR2), *Mus musculus* (Q91VH1, MouseAdpR1; Q8BQS5, MouseAdpR2), and *Rattus norvegicus* (Q6P746, Rat AdipR1; Q3YAF7, Rat AdipR2) by MUltiple Sequence Comparison by Log-Expectation (MUSCLE),. (*) indicates positions that have a single, fully conserved residue. (:) indicates conservation between groups of strongly similar properties. (.) indicates conservation between groups of weakly similar properties. The conserved residues for zinc binding site, the 3×His and Asp residues and a Ser residue, are shown in red. The seven transmembrane domain regions are marked by upper lines and yellow.

A_gambAdpR MSSLRYEMNDGIEGGIQQQPASSSGHKSALNAAPGDELFNNMMEADFSIRKRRVWAPEEV

HumanAdpR1 -------------MSSHKGSVVAQGNGAPASNREADTVELAELGPLLEEKGKRV------

MouseAdpR1 -------------MSSHKGSAGAQGNGAPSGNREADTVELAELGPLLEEKGKRA------

Rat AdpR1 -------------MSSHKGSAVAQGNGAPSSNREADTVELAELGPLLEEKGKRA------

HumanAdpR2 -MNEPTENRLGCSRTPEPDIRLRKGHQLDGTRRGDNDSHQGDLEPILEASVL--------

MouseAdpR2 -MNEPAKHRLGCTRTPEPDIRLRKGHQLDDTRGSNNDNYQGDLEPSLETPVC--------

Rat AdpR2 -MNEPTEHRLGCTRTPEPDIRLRKGHQLDDTRGGNNDNHHGDLEPSLETPVC--------

.*: : : . :.

A_gambAdpR SLASEDIDLLDDDDDLEEEEDDGVGCPLPSTPEDNQLLEAEMTEVLKAGVLSDEIDLGAL

HumanAdpR1 -------------IANPPKAEEEQTCPVPQEEEEEVRV---LTLPLQ-------------

MouseAdpR1 -------------ASSPAKAEEDQACPVPQEEEEEVRV---LTLPLQ-------------

Rat AdpR1 -------------ATSPAKAEEEQACPVPQEEEEEVRV---LTLPLQ-------------

HumanAdpR2 -------------SSHHKKSSEEHEYSDEAPQEDEGFM--GMSPLLQ-------------

MouseAdpR2 -------------SSYYENSPEEPECHDDNSQEDEGFM--GMSPLLQ-------------

Rat AdpR2 -------------SSYYENSPEELECHDDNSQEDEGFM--GMSPLLQ-------------

: : *:: : :: *:

A_gambAdpR AHNAAEQAEEFVRKVWEASWKVCHFKNLPAWLQDNDFLHKGHRPPLPSFSACFKSIFRIH

HumanAdpR1 AHHAMEKMEEFVYKVWEGRWRVIPYDVLPDWLKDNDYLLHGHRPPMPSFRACFKSIFRIH

MouseAdpR1 AHHAMEKMEEFVYKVWEGRWRVIPYDVLPDWLKDNDYLLHGHRPPMPSFRACFKSIFRIH

Rat AdpR1 AHHAMEKMEEFVYKVWEGRWRVIPYDVLPDWLKDNDYLLHGHRPPMPSFRACFKSIFRIH

HumanAdpR2 AHHAMEKMEEFVCKVWEGRWRVIPHDVLPDWLKDNDFLLHGHRPPMPSFRACFKSIFRIH

MouseAdpR2 AHHAMERMEEFVCKVWEGRWRVIPHDVLPDWLKDNDFLLHGHRPPMPSFRACFKSIFRIH

Rat AdpR2 AHHAMERMEEFVCKVWEGRWRVIPHDVLPDWLKDNDFLLHGHRPPMPSFRACFKSIFRIH

**:* *. **** ****. *.* .. ** **:***:* :*****:*** **********

Transmembrane domain 1 Transmembrane domain 2

A_gambAdpR TETGNIWTHLLGCVMFIGVAAYFLTRP--SFEIQLQEKLIFLTFFIGAIICLGFSFAFHT

HumanAdpR1 TETGNIWTHLLGFVLFLFLGILTMLRPNMYFMAPLQEKVVFGMFFLGAVLCLSFSWLFHT

MouseAdpR1 TETGNIWTHLLGFVLFLFLGILTMLRPNMYFMAPLQEKVVFGMFFLGAVLCLSFSWLFHT

Rat AdpR1 TETGNIWTHLLGFVLFLFLGILTMLRPNMYFMAPLQEKVVFGMFFLGAVLCLSFSWLFHT

HumanAdpR2 TETGNIWTHLLGCVFFLCLGIFYMFRPNISFVAPLQEKVVFGLFFLGAILCLSFSWLFHT

MouseAdpR2 TETGNIWTHLLGCVFFLCLGIFYMFRPNISFVAPLQEKVVFGLFFLGAILCLSFSWLFHT

Rat AdpR2 TETGNIWTHLLGCVFFLCLGIFYMFRPNISFVAPLQEKVVFGLFFLGAILCLSFSWLFHT

************ *:*: :. : ** * ****::* **:**::**.**: ***

Transmembrane domain 3 Transmembrane domain 4

A_gambAdpR LCCHSEMVGKLFSKLDYCGIALLIMGSFVPWLYYGFYCHYKHKLIYLTVVIVLGITSIIT

HumanAdpR1 VYCHSEKVSRTFSKLDYSGIALLIMGSFVPWLYYSFYCSPQPRLIYLSIVCVLGISAIIV

MouseAdpR1 VYCHSEKVSRTFSKLDYSGIALLIMGSFVPWLYYSFYCSPQPRLIYLSIVCVLGISAIIV

Rat AdpR1 VYCHSEKVSRTFSKLDYSGIALLIMGSFVPWLYYSFYCSPQPRLIYLSIVCVLGISAIIV

HumanAdpR2 VYCHSEGVSRLFSKLDYSGIALLIMGSFVPWLYYSFYCNPQPCFIYLIVICVLGIAAIIV

MouseAdpR2 VYCHSEGVSRLFSKLDYSGIALLIMGSFVPWLYYSFYCNPQPCFIYLIVICVLGIAAIIV

Rat AdpR2 VYCHSEGVSRLFSKLDYSGIALLIMGSFVPWLYYSFYCNPQPCFIYLIVICVLGIAAIIV

: **** *.. ******.****************.*** : :*** :: ****::**.

Transmembrane domain 5 Transmembrane

A_gambAdpR SLWDKFSQPNLRPLRAGVFMSFGLSGIIPAIHYVLMEGWFSKISQASLGWLILMGLLYIL

HumanAdpR1 AQWDRFATPKHRQTRAGVFLGLGLSGVVPTMHFTIAEGFVKATTVGQMGWFFLMAVMYIT

MouseAdpR1 AQWDRFATPKHRQTRAGVFLGLGLSGVVPTMHFTIAEGFVKATTVGQMGWFFLMAVMYIT

Rat AdpR1 AQWDRFATPKHRQTRAGVFLGLGLSGVVPTMHFTIAEGFVKATTVGQMGWFFLMAVMYIT

HumanAdpR2 SQWDMFATPQYRGVRAGVFLGLGLSGIIPTLHYVISEGFLKAATIGQIGWLMLMASLYIT

MouseAdpR2 SQWDMFATPQYRGVRAGVFVGLGLSGIIPTLHYVISEGFLKAATIGQIGWLMLMASLYIT

Rat AdpR2 SQWDMFATPQYRGVRAGVFVGLGLSGIIPTLHYVISEGFLKAATIGQIGWLMLMASLYIT

: ** *: *: * *****:.:****::*::*:.: **:.. : ..:**::**. :**

Domain 6 Transmembrane domain 7

A_gambAdpR GALFYALRVPERWFPGKCDIWFQSHQIFHVLVLVAAFVHYHGISEMAMYRVTV-GECDIP

HumanAdpR1 GAGLYAARIPERFFPGKFDIWFQSHQIFHVLVVAAAFVHFYGVSNLQEFRYGLEGGCT--

MouseAdpR1 GAGLYAARIPERFFPGKFDIWFQSHQIFHVLVVAAAFVHFYGVSNLQEFRYGLEGGCT--

Rat AdpR1 GAGLYAARIPERFFPGKFDIWFQSHQIFHVLVVAAAFVHFYGVSNLQEFRYGLEGGCT--

HumanAdpR2 GAALYAARIPERFFPGKCDIWFHSHQLFHIFVVAGAFVHFHGVSNLQEFRFMIGGGCS--

MouseAdpR2 GAALYAARIPERFFPGKCDIWFHSHQLFHIFVVAGAFVHFHGVSNLQEFRFMIGGGCT--

Rat AdpR2 GAALYAARIPERFFPGKCDIWFHFHQLFHIFVVAGAFVHFHGVSNLQEFRFMIGGGCT--

** :** *:***:**** ****: **:**::*:..****::*:*:: :* : * *

A_gambAdpR HQHPAISF

HumanAdpR1 -DDTLL--

MouseAdpR1 -DDSLL--

Rat AdpR1 -DDSLL--

HumanAdpR2 -EEDAL--

MouseAdpR2 -EEDAL--

Rat AdpR2 -EEDAL--

: :
