# Supplementary material for: Signaling between mammalian adiponectin and a mosquito adiponectin receptor reduces Plasmodium transmission
Source: mBio. 2023 Dec 11;15(1):e02257-23. doi: 10.1128/mbio.02257-23 (PMC10790699; doi:10.1128/mbio.02257-23)
Supplement: Table S2 — Anopheles gambiae genes that are significantly changed (adjusted P < 0.05) in mosquitoes which the adiponectin receptor gene has been silenced, compared with control mosquitoes. [file mbio.02257-23-s0005.docx]

**Table S2. *Anopheles gambiae* genes that are significant changed (adjusted P<0.05) in mosquitoes which the adiponectin receptor gene has been silenced, compared with control mosquitoes.** Both groups of mosquitoes have taken a blood meal from mice infected with *Plasmodium berghei*.

| **Gene ID** | **log2FoldChange** | **padj** | **Gene name** |  |
| --- | --- | --- | --- | --- |
| AGAP000179 | 4.963703323 | 0.003146 | Amidophosphoribosyltransferase |  |
| AGAP000182 | 3.380140653 | 0.042592 |  |  |
| AGAP000211 | 4.981250801 | 0.003146 | triacylglycerol lipase |  |
| AGAP000573 | 3.723027785 | 0.019337 | CLIP-domain serine protease | CLIPC4 |
| AGAP000779 | 2.544705191 | 0.024318 | zinc finger homeobox protein 1/2 |  |
| AGAP000955 | 4.171563607 | 0.020411 |  |  |
| AGAP000996 | 2.85237817 | 0.01598 | ZZ-type domain-containing protein |  |
| AGAP000999 | 2.595988127 | 0.041791 | TOLL-like receptor 5A | TOLL5A |
| AGAP001076 | 4.494275575 | 0.004306 | cytochrome P450 | CYP4G16 |
| AGAP001124 | 4.996856176 | 0.027189 | Aminomethyltransferase |  |
| AGAP001127 | 5.667361653 | 0.011466 | P37NB protein |  |
| AGAP001376 | 3.149431252 | 0.003146 | serine protease inhibitor (serpin) 17 | SRPN17 |
| AGAP001582 | 5.067778803 | 0.001994 |  |  |
| AGAP001662 | 3.229732534 | 0.001734 |  |  |
| AGAP001769 | 4.681230637 | 0.0175 | beat protein |  |
| AGAP001826 | 6.097788612 | 1.33E-05 | lipophorin | Lp |
| AGAP001899 | 4.212767845 | 0.038044 | fatty acid synthase |  |
| AGAP001989 | 5.191832349 | 0.001994 |  |  |
| AGAP002422 | 3.214113833 | 0.043281 | CLIP-domain serine protease | CLIPD1 |
| AGAP002534 | 1.676455155 | 0.00602 | dihydropteridine reductase |  |
| AGAP002582 | -4.844450678 | 0.025846 |  |  |
| AGAP002594 | 3.74921372 | 4.04E-05 | apolipoprotein D |  |
| AGAP002632 | 5.16272924 | 0.019405 |  |  |
| AGAP002799 | -7.216905256 | 0.019337 | 3-glucan binding protein | GNBPB3 |
| AGAP002830 | 3.846034329 | 0.005186 |  |  |
| AGAP003007 | -3.733618923 | 0.002676 |  |  |
| AGAP003057 | 3.353425915 | 0.001481 | CLIP-domain serine protease | CLIPB8 |
| AGAP003160 | -1.335469964 | 0.001447 | MAP kinase interacting serine/threonine kinase |  |
| AGAP003246 | 5.721494116 | 0.004213 | CLIP-domain serine protease | CLIPB2 |
| AGAP003250 | 4.26677409 | 0.006274 | CLIP-domain serine protease | CLIPB4 |
| AGAP003474 | 4.930412442 | 0.003146 |  |  |
| AGAP003516 | 1.51762794 | 0.046611 |  | NIT8492 |
| AGAP004017 | 3.248763944 | 0.018334 |  |  |
| AGAP004154 | 6.536172571 | 0.01581 | jagged |  |
| AGAP004450 | 2.958894048 | 0.0175 |  |  |
| AGAP004486 | -2.001456606 | 1.33E-05 | adiponectin receptor |  |
| AGAP004794 | 4.297744268 | 0.045297 | pancreatic triacylglycerol lipase |  |
| AGAP004977 | 3.763736362 | 0.017076 | prophenoloxidase 6 | PPO6 |
| AGAP004978 | 4.825740097 | 0.003408 | prophenoloxidase 9 | PPO9 |
| AGAP004981 | 4.097772104 | 0.007963 | prophenoloxidase 4 | PPO4 |
| AGAP005009 | 5.256273488 | 0.006274 | Pyrroline-5-carboxylate reductase |  |
| AGAP005340 | 3.86378045 | 0.003717 |  |  |
| AGAP005693 | 4.056599559 | 0.046731 | leucine-rich immune protein (Coil-less) | LRIM17 |
| AGAP005985 | 7.71450767 | 0.009651 | fatty acyl-CoA reductase 2 |  |
| AGAP006192 | 4.231065514 | 0.02365 | chymotrypsin B chain C |  |
| AGAP006258 | 4.574755914 | 1.17E-05 | prophenoloxidase 2 | PPO2 |
| AGAP006275 | 4.548060138 | 0.018641 |  |  |
| AGAP006278 | 4.187751 | 0.002337 |  |  |
| AGAP006743 | 3.98207435 | 0.046139 |  |  |
| AGAP006910 | 5.034511003 | 0.001147 | serine protease inhibitor (serpin) 3 | SRPN3 |
| AGAP006911 | 3.740399174 | 0.046611 | serine protease inhibitor (serpin) 2 | SRPN2 |
| AGAP007043 | 4.733138313 | 0.021495 |  |  |
| AGAP007045 | 3.923004208 | 0.001734 | leucine-rich immune protein (TM) | LRIM15 |
| AGAP007107 | 1.629503902 | 0.003089 | DnaJ homolog subfamily B member 4 |  |
| AGAP007123 | 6.438628655 | 0.005851 | sarcosine dehydrogenase |  |
| AGAP007312 | 3.788752568 | 0.001643 |  |  |
| AGAP007647 | 5.25669988 | 0.001643 |  |  |
| AGAP008013 | 3.634870319 | 0.010586 |  |  |
| AGAP008060 | 3.744964958 | 0.021779 | imaginal disc growth factor 2 | IDGF2 |
| AGAP008061 | 5.790544033 | 0.000396 | imaginal disc growth factor 4 | IDGF4 |
| AGAP008279 | 3.714792534 | 0.006274 | D7 long form salivary protein | D7L2 |
| AGAP008338 | -5.786301691 | 0.029699 |  |  |
| AGAP008596 | 3.744981979 | 0.0175 | long-chain-fatty-acid--CoA ligase ACSBG |  |
| AGAP008807 | 4.818298513 | 0.000864 |  |  |
| AGAP009128 | 4.718198055 | 0.028167 | mitochondrial carnitine/acylcarnitine carrier protein | |
| AGAP009146 | 4.978184518 | 0.001134 |  |  |
| AGAP009217 | 7.268812328 | 0.000179 | CLIP-domain serine protease | CLIPB12 |
| AGAP009462 | -2.492076816 | 0.038044 |  |  |
| AGAP009493 | 2.575871028 | 0.001465 | nicotinic acetylcholine receptor subunit alpha 9 |  |
| AGAP009762 | 2.345601326 | 0.012508 |  |  |
| AGAP010531 | 3.826883615 | 0.012142 | fibrinogen-related protein 7 |  |
| AGAP010546 | 5.211960697 | 0.01489 |  |  |
| AGAP010547 | 4.862177372 | 0.004243 |  |  |
| AGAP010812 | 3.29913882 | 0.0175 | thioester-containing protein 4 | TEP4 |
| AGAP010968 | 5.038938783 | 0.006152 | CLIP-domain serine protease | CLIPA9 |
| AGAP011183 | 4.203185437 | 0.024486 |  |  |
| AGAP011223 | 3.389601381 | 0.019337 | Fibrinogen C-terminal domain-containing protein |  |
| AGAP011562 | 5.457892791 | 0.006361 | outer segment 4 | Oseg4 |
| AGAP011604 | 4.434810967 | 1.33E-05 |  |  |
| AGAP011765 | 5.030194209 | 0.020411 | Spondin-1 |  |
| AGAP011780 | 4.931639261 | 0.010586 | CLIP-domain serine protease | CLIPA4 |
| AGAP011787 | 7.850397655 | 0.012184 | CLIP-domain serine protease | CLIPA5 |
| AGAP011788 | 3.969782465 | 0.025405 | CLIP-domain serine protease | CLIPA14 |
| AGAP011870 | 2.074352288 | 0.007445 | matrix metalloprotease 2 | MMP2 |
| AGAP012000 | 3.359804849 | 0.019337 | fibrinogen and fibronectin |  |
| AGAP012532 | -8.390996957 | 0.000207 |  |  |
| AGAP012571 | 4.72077236 | 0.003554 |  |  |
| AGAP013094 | 7.500905383 | 0.018097 | Elongation of very long chain fatty acids protein |  |
| AGAP013117 | 3.889264874 | 0.020411 |  |  |
| AGAP013166 | 3.478554869 | 0.02365 | chitinase | Cht5-1 |
| AGAP013218 | 2.340898046 | 0.006834 | sodium-independent sulfate anion transporter |  |
| AGAP013231 | 3.913223654 | 0.026175 | Nitrilase homolog 2 |  |
| AGAP013443 | 3.742565704 | 0.006299 | Plasma glutamate carboxypeptidase |  |
| AGAP013509 | 3.662786841 | 0.019337 | carboxylesterase clade H, member 1 |  |
