# Supplementary material for: Signaling between mammalian adiponectin and a mosquito adiponectin receptor reduces Plasmodium transmission
Source: mBio. 2023 Dec 11;15(1):e02257-23. doi: 10.1128/mbio.02257-23 (PMC10790699; doi:10.1128/mbio.02257-23)
Supplement: Table S3 — Primers used in the studies. [file mbio.02257-23-s0006.docx]

**Table S3. Primers used in the studies.**

|  | **Primer Name** | **Sequence 5'-3'** |
| --- | --- | --- |
| **dsRNA synthesis** | |  |
| ds*AdpR* | F | TAATACGACTCACTATAGGGAGGGTGGCATCCAGCAGCAGCCAGC |
|  | R | TAATACGACTCACTATACCCAGTCCATATGTTGCCGGTTTCGGTGTG |
| ds*Gluc* | F | TAATACGACTCACTATAGGGCCACCGAGAACAACGAAGAC |
|  | R | TAATACGACTCACTATAGGGCCCTTGATCTTGTCCACCTG |
| ds*Lp* | F | TAATACGACTCACTATAGGGCCCAGAAGATGGGCGAACAGTC |
|  | R | TAATACGACTCACTATAGGGCCGAGCGATTCGGGATGCAG |
| **Protein Expression** | |  |
| For GFP fusion | For | GGCGCGGTACCCGTTGTCGTTGCGTGCGTATCAAC |
|  | Rev | GGCGCGGATCCCGCTAATAGCAGGATGCT |
| **RT-PCR for *A gambiae*** | |  |
| *AdpR* | F | TCGCAGCCAAATCTGAGAC |
|  | R | CAGGATAAGCCATCCCAGAC |
| *Actin* | F | GAAGGCTAACCGCGAGAAGATG |
|  | R | CGCCGGAGTCCAGCACGATA |
| *AGAP008838* | F | TGAAGGATGCATGGACTTTG |
|  | R | ATCGAGTCGCTTGAGTTCGT |
| *AGAP012532* | F | AGGTGTTTTGATTCGCCAAC |
|  | R | ACAACAACGTGCCAGTACCA |
| *AGAP013094* | F | CTGGAGCTGTGAAACGATCA |
|  | R | AGCAGCTACCATTCCAGCAT |
| *AGAP002799* | F | ACGGAACAGTTCGGATCAAC |
|  | R | GCATCGATCTGTGTGACGAC |
| *AGAP011787* | F | GAACGATGTGGCACTGCTAA |
|  | R | ACCTGGTACTTGCCCTCCTT |
| *AGAP009217* | F | GCCTGGCTTAGATTTGCTTG |
|  | R | TTATGCAGAACGCAGTCGTC |
| *AGAP008061* | F | TAAAGTGTGGCACGGATTCA |
|  | R | TATCCTTCAGCAGGGGAATG |
| *Lipophorin* | F | CAGCCAGGATGGTGAGCTTAA |
|  | **R** | CACCAGCACCTTGGCGTT |
| **RT-PCR for *P. berghei*** |  |  |
| *Pb 18s* | F | AAGCATTAAATAAAGCGAATACATCCTTAC |
|  | R | GGAGATTGGTTTTGACGTTTATGTG |
|  |  |  |
